# Immunosenescence and Bone Homeostasis: From Mechanisms of Homeostasis Disruption to Therapeutic Opportunities in Age-Related Skeletal Disorders

**DOI:** 10.3390/ijms27104322

**Published:** 2026-05-12

**Authors:** Fuhan Jiang, Bowen Dong, Yijue Wang, Yi Xiong

**Affiliations:** 1 State Key Laboratory of Oral Diseases & National Center for Stomatology & National Clinical Research Center for Oral Diseases, West China Hospital of Stomatology, Sichuan University, Chengdu 610041, China; 2 Department of Oral Implantology, West China Hospital of Stomatology, Sichuan University, Chengdu 610041, China

**Keywords:** immunosenescence, aging, bone homeostasis, age-related bone diseases, senescent bone niche

## Abstract

The progressive decline in immune function during aging, termed immunosenescence, is increasingly recognized as a critical driver of skeletal fragility and impaired bone regeneration. This age-associated phenomenon—driven by thymic involution, inflammaging, and the accumulation of senescent immune cells—disrupts bone homeostasis primarily through the establishment of a pro-inflammatory milieu, wherein senescence-associated secretory phenotype (SASP) factors directly reprogram the function and fate of mesenchymal stem cells, osteoblasts, osteoclasts, and chondrocytes. Clinically, this immune-driven disruption of the bone microenvironment manifests across a spectrum of age-related skeletal disorders—including osteoporosis and osteoarthritis as prototypes of systemic and local bone loss, respectively, as well as delayed fracture healing, intervertebral disc degeneration, and periodontitis as paradigms of impaired regenerative and defensive responses. Despite advances in osteoimmunology revealing bidirectional immune-bone interactions, the mechanistic links between senescent immune cells and bone pathophysiology remain incompletely defined, presenting a significant barrier to therapeutic innovation. Herein, we synthesize current evidence to elucidate how immunosenescence, through the dysfunction of both innate and adaptive immunity, progressively dismantles bone homeostasis. We critically evaluate current challenges in dissecting the relative contributions of immunological memory accumulation versus fundamental aging processes to skeletal decline. We identify key knowledge gaps and propose strategic research directions, including longitudinal human immunophenotyping studies and innovative organoid-immune aging models. Such approaches hold the potential to transform the therapeutic landscape of age-related skeletal diseases by enabling precision interventions that target specific immunosenescence pathways to rejuvenate the aging skeleton.

## 1. Introduction

The progressive decline of immune function during aging—termed immunosenescence—is increasingly recognized as a key determinant of health span, contributing to infections, malignancies, and chronic diseases [1,2]. Among affected systems, the skeleton is particularly vulnerable. Emerging evidence establishes immunosenescence as a pivotal driver of bone homeostasis disruption, a process essential to skeletal integrity throughout life [3]. The clinical burden of age-related skeletal disorders is substantial. Osteoporosis affects over 200 million individuals worldwide, with one in three women and one in five men over 50 years of age experiencing an osteoporotic fracture in their lifetime [4]. Osteoarthritis impacts approximately 528 million people globally, with population aging serving as the primary driver of its increasing prevalence [5]. Low back pain, primarily attributed to intervertebral disc degeneration, affects about 40% of the global population, and in China, up to 90% of individuals over 60 years of age suffer from this condition [6]. Severe periodontitis affects over 1.07 billion people worldwide, a number projected to exceed 1.5 billion by 2050 [7]. Collectively, these conditions impose a staggering socioeconomic burden, with population aging contributing an estimated US $96.0 billion to global healthcare costs for musculoskeletal (MSK) disorders in 2021 alone—a figure driven primarily by osteoporosis, osteoarthritis, and spinal conditions [8].

Mechanistically, immunosenescence disrupts bone homeostasis by establishing a pro-inflammatory microenvironment within bone marrow, a state intimately linked to “inflammaging”—a chronic, low-grade inflammation driven by senescent immune cells that forms a self-reinforcing vicious cycle with immunosenescence [9,10]. The bone marrow, as a highly immunologically active niche, is particularly susceptible to this cycle, wherein accumulating senescent immune cells amplify local inflammation and disrupt skeletal homeostasis [11]. Senescent immune cells (macrophages, T cells, B cells) acquire a senescence-associated secretory phenotype (SASP), releasing cytokines and proteases that impair bone-resident cells [12]. Consequently, mesenchymal stem cells (MSCs) exhibit diminished osteogenesis, osteoblasts show reduced bone formation, osteoclasts become hyperactivated, and chondrocytes undergo accelerated degradation—collectively leading to bone loss and compromised regeneration. Clinically, this immune-driven deterioration manifests across age-related skeletal disorders. Osteoporosis and osteoarthritis represent prototypes of systemic and local bone loss, wherein immunosenescence fuels resorption-formation imbalance [13,14]. Under injury or microbial challenge, compromised immunity exacerbates pathology: delayed fracture healing [15], accelerated disc degeneration [16] and amplified periodontal bone loss [17].

Despite advances in osteoimmunology, how senescent immune cells actively drive bone pathophysiology remains ill-defined. Distinguishing intrinsic aging from immunological memory effects, and understanding osteocytes as active participants in immune dialogue, remain critical gaps [12]. These barriers hinder targeted therapy development. Here, we synthesize evidence elucidating how immunosenescence progressively dismantles bone homeostasis. We propose a “senescent bone niche” framework integrating disparate observations into a cohesive disease model. Finally, we identify key questions and strategic directions to pave the way for precision interventions rejuvenating the aging skeleton.

## 2. Bone Homeostasis and Immunosenescence

### 2.1. Bone Homeostasis

Bone homeostasis is maintained by the dynamic balance between osteoclast-mediated bone resorption and osteoblast-driven bone formation—a continuous remodeling cycle that replaces old bone with new tissue to preserve skeletal integrity throughout life [18]. This process, initiated by resorption followed by formation, enables the repair and maintenance of bone tissue and its surrounding microenvironment [19].

**Figure 1:**
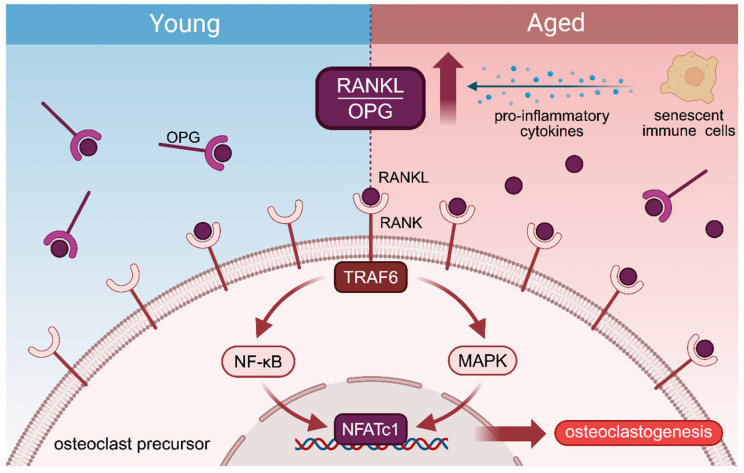
RANKL/RANK/OPG signaling in osteoclastogenesis under physiological and aging conditions. Red arrow indicates an increase.

Multiple signaling pathways act in concert during bone formation and resorption. Among them, the RANK/RANKL/OPG axis is particularly notable for its central role in osteoclast regulation and its close interplay with the immune system [20]. Receptor activator of nuclear factor-κB ligand (RANKL), produced by osteoblasts, osteocytes, and bone marrow stromal cells, binds to receptor activator of nuclear factor-κB (RANK) on osteoclast precursors, recruiting TNF receptor-associated factor 6 (TRAF6) to activate downstream cascades (e.g., nuclear factor-κB (NF-κB), mitogen-activated protein kinase (MAPK)) [21], ultimately inducing nuclear factor of activated T cells, cytoplasmic 1 (NFATc1)—the master transcription factor for osteoclastogenesis [22]. Osteoprotegerin (OPG), a decoy receptor secreted by osteoblasts, competitively binds RANKL to prevent RANK engagement, thereby restraining excessive osteoclast activity [23]. The RANKL/OPG ratio thus serves as a critical determinant of net bone resorption activity (Figure 1).

Aging progressively disrupts this equilibrium. Cortical bone becomes porous, trabecular bone loses density, and the mineralized collagen network weakens—structural declines that collectively impair bone strength and toughness [24,25,26]. At the cellular level, these changes reflect a fundamental imbalance: the osteogenic potential of MSCs diminishes, while osteoclast activity becomes dysregulated, culminating in progressive bone loss and impaired regenerative capacity [27,28]. In the aging skeleton, an increased RANKL/OPG ratio, driven in part by pro-inflammatory cytokines from senescent immune cells, shifts the balance toward uncontrolled osteoclastogenesis [29].

While the RANK/RANKL/OPG axis provides a well-established framework for understanding osteoclast regulation, it is increasingly evident that bone homeostasis is shaped by a far more complex network of interactions extending beyond this single pathway. These include direct cell-to-cell contact [30], paracrine signaling via extracellular vesicles [31], metabolic reprogramming of immune and skeletal cells [32], and epigenetic regulation [33], all of which have emerged as critical mediators of immunosenescence-driven skeletal deterioration. Elucidating how immunosenescence contributes to this deterioration is essential for identifying therapeutic targets that address the underlying drivers of skeletal aging.

RANKL binds to RANK on osteoclast precursors, recruiting TRAF6 and activating NF-κB and MAPK pathways, which induce NFATc1 and promote osteoclastogenesis. OPG competitively binds RANKL to inhibit this process. Under aging conditions, pro-inflammatory cytokines released by senescent immune cells increase the RANKL/OPG ratio, enhancing RANK signaling and osteoclast formation. Created in BioRender. Dong, B. (2026). Retrieved from https://BioRender.com/cqvwkp8 (accessed on 3 April 2026).

### 2.2. Immunosenescence

**Figure 2:**
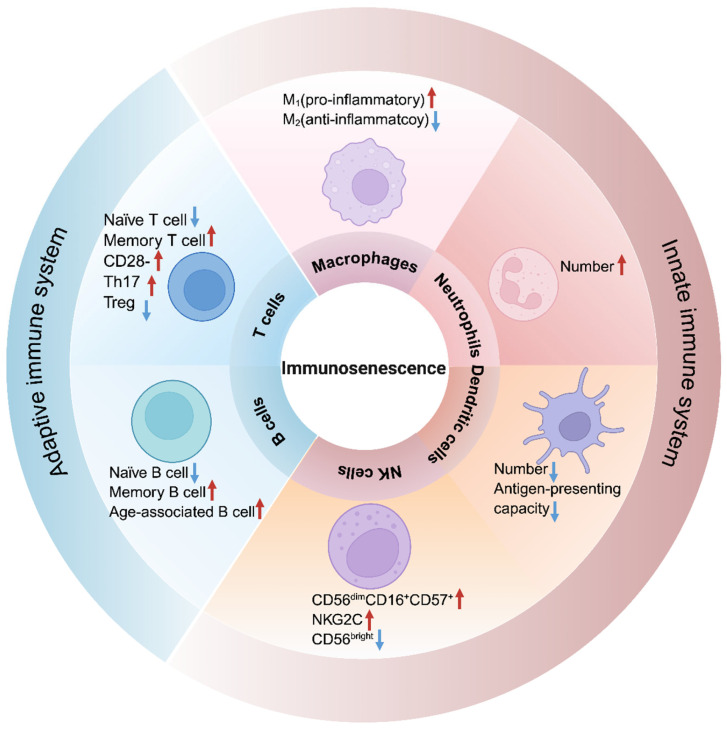
Overview of immunosenescence-induced changes in immune cell subsets critical for bone homeostasis. Immunosenescence alters the profiles of T cells, B cells, dendritic cells, natural killer cells (NK cells), myeloid-derived suppressor cells (MDSCs), and macrophages, leading to shifts in their abundance and functionality, which significantly impact the maintenance of bone homeostasis. Red arrows indicate upregulation or increase; blue arrows indicate downregulation or decrease. Created in BioRender. Dong, B. (2026). Retrieved from https://BioRender.com/br6dw36 (accessed on 3 April 2026).

The immune system serves as a critical cornerstone in preserving health. During aging, the immune system undergoes progressive functional decline, a phenomenon termed immunosenescence [1], which not only contributes to susceptibility to infections, tumors, and autoimmune diseases [34], but also significantly impacts systemic and skeletal health through multiple cellular mechanisms (Figure 2 and Table 1). Immunosenescence does not uniformly affect all immune cells; rather, it induces distinct phenotypic and functional alterations across different immune cell types, each with unique implications for the bone microenvironment.

#### 2.2.1. Macrophages

Aging profoundly affects macrophages through multiple mechanisms. Senescent cells can actively drive macrophages into a senescent state [51], while macrophage polarization—the balance between pro-inflammatory (M1) and anti-inflammatory (M2) phenotypes—becomes dysregulated with age [52]. This M1/M2 imbalance contributes to a chronic, low-grade inflammatory microenvironment (inflammaging) that can disrupt bone homeostasis [12]. Additionally, aged macrophages exhibit impaired antigen-presenting function, reflected by downregulated expression of major histocompatibility complex (MHC) class II molecules and toll-like receptors (TLRs) [12].

#### 2.2.2. T Cells

Immunosenescence drives alterations in the absolute/relative abundance of specific immune cell populations. During aging, T cell subsets exhibit significant changes, characterized by the decrease in naïve T cells and the increase in CD28^−^ memory T cells, which are often termed “immunosenescent” [1]. CD28^−^ T cells display hallmark features associated with cellular aging, including shortened telomere length, impaired antigen-induced proliferative capacity, elevated production of pro-inflammatory cytokines, and downregulated expression of molecules linked to cytotoxic activity, such as perforin, granzyme B, and senescence-associated β-galactosidase (SA-β-gal) [53]. Notably, T cell immunosenescence is not entirely cell-autonomous, aged B cells, via MHC class II-dependent mechanisms, can actively promote the differentiation of naïve CD4^+^ T cells into immunosenescent subsets, thereby exacerbating systemic immune decline and compromising health span [54]. However, the classification of these cells as “senescent” requires nuance. Single-cell analyses have revealed that some immune cells with senescent characteristics exhibit high Ki67 expression, challenging the conventional notion that senescence equates to irreversible growth arrest [55]. This complexity points to a distinct “immunosenescent” phenotype that may not fully conform to classical senescence paradigms. A more precise definition of T cell senescence—integrating molecular hallmarks with immune-specific functional phenotypes—is therefore essential to accurately assess its contribution to age-related bone pathology.

A functional distinction between T cell senescence and exhaustion is essential for accurately interpreting their roles in age-related bone pathology. While both states are characterized by diminished proliferative capacity, they arise from fundamentally distinct mechanisms. Senescence is primarily driven by intrinsic cellular stressors such as DNA damage and replicative arrest, often accompanied by pro-inflammatory SASPs [56]. In contrast, exhaustion is induced by chronic antigen stimulation and is defined by sustained expression of co-inhibitory receptors (e.g., programmed cell death protein 1 (PD1), cytotoxic T lymphocyte-associated antigen 4 (CTLA4)), and remains partially reversible upon checkpoint blockade [57]. These two states are frequently conflated in the literature, as markers such as killer cell lectin-like receptor subfamily G member 1 (KLRG1) and CD57 are used in both contexts [58,59], yet they can exhibit discordant expression patterns within the same patient cohort [60]. This conflation underscores the need for a precise functional delineation of these distinct T cell states within the bone niche. This review focuses on the cell-intrinsic mechanisms of T cell senescence and their implications for age-related bone disorders.

#### 2.2.3. B Cells

Aging impairs B cell development and function at multiple levels. Large-scale 3D chromatin reorganization in aged progenitor B cells silences key regulators such as early B cell factor 1 (Ebf1), providing an epigenetic basis for the decline in lymphopoiesis [49]. These changes manifest in the B cell pool as a contraction of naïve subsets [45] and an expansion of pro-inflammatory aging-associated B cells (ABCs) that secrete TNFα and inhibit the generation of young pro-B cells [48]. Single-cell analyses have further identified novel senescent B cell subsets in humans that express canonical SASP components like S100A8/A9 [47].

Critically, the bone marrow niche itself deteriorates with age: the loss of trabecular bone physically displaces B cell progenitors from supportive microenvironments, a process exacerbated by local TLR4 signaling [61]. Beyond local factors, systemic endocrine signals, such as growth hormone signaling, also contribute to age-associated B cell dysregulation [62]. Collectively, these age-related changes in B cells—both intrinsic and niche-driven—contribute to the disruption of bone homeostasis [63].

## 3. Immunosenescence and Its Impact on Bone-Related Cells

### 3.1. Mesenchymal Stem Cells

Mesenchymal stem cells, first identified in bone marrow stroma, possess multilineage differentiation potential giving rise to chondrocytes, adipocytes, and osteoblasts—playing indispensable roles in maintaining bone homeostasis [64,65]. Their functionality is tightly regulated by the bone marrow niche, a specialized microenvironment comprising vascular networks, stromal cells, and immune cells that sustain stem cell properties and govern their survival, activation, and migration [66,67].

Within this niche, macrophages are key regulators of MSC fate. M2 macrophages promote MSC recruitment and osteogenic differentiation through secretion of growth factors [68]. However, with age, macrophages shift toward the pro-inflammatory M1 phenotype, disrupting this crosstalk and impairing MSC osteogenesis [69]. This age-related macrophage dysregulation has emerged as a therapeutic target. In murine models, strategies that promote M2 polarization—such as dimethyl itaconate-releasing materials [70], rapamycin-loaded nanocarriers [71], and magnesium-based scaffolds that activate Nrf2 signaling [72]—have been shown to enhance MSC osteogenic differentiation and improve bone defect repair. Collectively, these studies demonstrate that immunomodulation of macrophage polarization represents a promising approach to preserve MSC function in the aging skeleton.

Beyond macrophages, senescent immune cells—including macrophages and neutrophils—directly compromise the MSC niche through the secretion of SASP factors [73]. Emerging evidence has revealed that chronic inflammation adversely affects the Wnt/β-catenin pathway that is essential for MSCs osteogenesis and bone matrix mineralization [74]. Additionally, fundamental research suggests that targeting the Wnt/NF-κB axis holds therapeutic potential. For instance, in mouse models of osteoporosis and aging, the Wnt ligand Wnt4 was shown to inhibit NF-κB signaling in osteoclast precursors and macrophages, thereby reducing inflammation and bone loss while promoting bone formation [75]. More specifically, Li et al. identified grancalcin (GCA) as a critical SASP factor secreted by senescent macrophages and neutrophils that binds to the Plexin B2 receptor on MSCs, suppressing osteogenesis and promoting adipogenesis [30]. Importantly, neutralizing GCA antibodies restores skeletal health in aged mice [30]. These findings highlight the therapeutic potential of targeting specific SASP factors, rather than broadly inhibiting inflammation, to rejuvenate the aged MSC niche.

Despite these advances, the remarkable heterogeneity of the SASP—and the fact that many SASP factors overlap with general inflammatory mediators—poses a significant challenge. Identifying which SASP components are both specific to senescence and causally linked to MSC dysfunction remains a critical knowledge gap. Filling this gap will be essential for developing precision interventions that selectively neutralize pathogenic SASP factors without compromising beneficial immune functions.

### 3.2. Osteoblasts

Osteoblasts, the principal bone-forming cells derived from MSCs, play a central role in skeletal homeostasis through their dual function of extracellular matrix synthesis and mineralization. These cells produce type I collagen, which serves as a structural scaffold for hydroxyapatite crystallization, thereby driving bone mineralization [19]. Importantly, osteoblasts coordinate with osteoclasts in a tightly coupled manner to maintain bone remodeling equilibrium, with this balance being crucial for skeletal integrity throughout life [76].

The differentiation and activity of osteoblasts are intricately regulated by immune cells, primarily through the canonical Wnt/β-catenin signaling pathway [77]. Mechanistically, research in mice has shown that regulatory T cells (Tregs) stimulate the formation of the NFAT1/SMAD3 transcriptional complex in CD8^+^ T cells [78]. Then, the stimulated CD8^+^ T cells secret Wnt10b [78], triggering β-catenin stabilization through binding LRP5 on osteoblast precursors, ultimately inducing the expression of key osteogenic transcription factors such as Runx2, Osterix, and Dlx5 [79], and promoting osteoblast differentiation and bone formation [78].

Immunosenescence profoundly disrupts this regulatory network through multiple mechanisms. Senescent T cells are closely related to bone regeneration. One study showed that the senescent CD8^+^CD28^−^CD57^+^ T cell subset could inhibit osteoblast differentiation and impair the survival of MSCs by secreting large amounts of pro-inflammatory cytokines (TNFα, IL17A, and IFNγ), resulting in a decreased number and function of osteoblasts [41]. In addition, the osteogenic-supportive function of Tregs is compromised with age. A recent study in aged mice found that senescent Tregs an exhibit impaired ability to induce osteogenesis due to reduced secretion of progranulin (PGRN), a key factor that activates the osteogenic phosphoinositide 3-kinase (PI3K)/protein kinase B (AKT) pathway in bone-forming precursors [80]. This finding was further supported by Yu et al., who found that iPTH-mediated expansion of Treg populations enhanced bone formation. Conversely, Treg blockade resulted in reduced proliferation, differentiation, and longevity of osteoblasts in animal models, along with impaired CD8^+^ T cell-mediated bone formation [81]. This evidence indicates that pharmacological augmentation of Tregs could serve as an innovative anabolic approach for bone regeneration, whereas the Treg expansion induced by intermittent parathyroid hormone (iPTH) might have therapeutic potential for inflammatory disorders and transplantation medicine.

Beyond T cells, megakaryocyte (MK) dysfunction also significantly impairs bone formation. Studies in murine models indicate that while healthy MKs enhance osteoblast proliferation via TGFβ secretion, senescent MKs show diminished transforming growth factor (TGF)β production, consequently suppressing osteoblast differentiation and exacerbating age-related bone loss [82]. Therefore, Tregs and CD8^+^ T cells disrupted by immunosenescence play essential roles in osteoblast differentiation, affecting bone homeostasis through inflammatory cytokine secretion and impaired Wnt10b production, while megakaryocyte dysfunction further exacerbates age-related bone loss via diminished TGFβ output.

### 3.3. Osteocytes

During bone remodeling, osteoblasts follow distinct fates: while a subset of osteoblasts undergo apoptosis, others become embedded within the bone matrix and terminally differentiate into osteocytes [19]. These cells establish an extensive dendritic network that permeates the mineralized matrix, serving as both structural components and master regulators of bone homeostasis [19]. Osteocytes function as mechanosensors that translate mechanical stimuli into biochemical signals, and as endocrine regulators that coordinate osteoblast and osteoclast activity through paracrine signaling [83].

Osteocyte senescence drives increased production of osteocyte-derived RANKL, which is a key driver of age-related bone loss [84]. Beyond RANKL, other age-related dysfunctions, including apoptosis and impaired mechanotransduction, also contribute to uncoupling bone formation from resorption [85]. Collectively, these alterations contribute to a microenvironment that promotes bone aging and fragility fractures. While oxidative stress and DNA damage are established inducers of osteocyte senescence [86], how these cell-intrinsic inducers are influenced by the aged systemic environment, particularly by inflammaging, remains poorly understood.

Emerging evidence implicates immune dysfunction in osteocyte senescence. Recent transcriptomic analyses in murine models have revealed that pro-inflammatory cytokines and immune dysfunction can induce osteocyte senescence [87]. This relationship is bidirectional: senescent osteocytes secrete SASP factors that exacerbate local inflammation [86], while SASP from external senescent cells impairs osteocyte function. Specifically, conditioned medium from senescent periodontal ligament cells disrupts the osteocyte dendritic network [88], and SASP from senescent osteoblasts or osteocytes impairs osteocyte mechanotransduction via IL6-mediated downregulation of P2X7 expression [89]. Although senescent immune cells are known to accumulate in the senescent bone niche, direct evidence that they impair osteocyte function is lacking, particularly regarding the roles of specific immune cell subsets and their secretory profiles. Nevertheless, evidence from murine studies demonstrates that TNFα directly upregulates RANKL mRNA expression in primary osteocytes and increases the proportion of RANKL-positive osteocytes, an effect mediated through the MAPK and NF-κB pathways [90]. Consistent with these findings, inflammaging, which is characterized by elevated levels of TNFα and other SASP factors, may similarly enhance osteocyte-derived RANKL production, thereby contributing to age-related bone loss.

Given the osteocyte’s central role in orchestrating bone remodeling, understanding how senescent immune cells compromise osteocyte function may provide new insights into age-related skeletal fragility.

### 3.4. Osteoclasts

Osteoclasts, derived from the hematopoietic lineage, are large multinucleated cells that tightly adhere to bone surfaces and mediate bone matrix resorption [19]. The differentiation and activation of osteoclasts are governed by two essential cytokines: macrophage colony-stimulating factor (M-CSF), which drives hematopoietic stem cells into the monocyte/macrophage lineage [91], and RANKL, which binds to RANK to activate NF-κB and NFATc1, inducing osteoclast-specific gene expression [92].

Immunosenescence regulates osteoclast differentiation through multiple immune cell subsets. T helper 17 cells (Th17) serve as major sources of RANKL, while Tregs typically suppress osteoclastogenesis [93]. With age, senescent CD4^+^CD28^−^ T cells accumulate and produce more RANKL, exhibiting enhanced ability to promote osteoclast differentiation [29]. Intriguingly, Fessler et al. described a novel CD4^+^CD28^−^FoxP3^+^ T cell subset that combines features of both Tregs and senescent T lymphocytes, potentially representing a terminal differentiation state of Tregs [94]. These cells display a pro-inflammatory phenotype characterized by high SA-β-gal activity and increased secretion of TNFα, IFNγ, and IL17A, which may contribute to age-related bone loss [94]. For instance, in both arthritis and periodontitis, senescent T cell subsets (e.g., exFoxp3^+^ Th17 cells) that express markers of cellular senescence (such as KLRG1) produce IL17A and RANKL, thereby promoting osteoclast differentiation and driving pathological bone resorption [95,96].

During aging, macrophages fail to polarize to M2 phenotype, resulting in chronic low-grade inflammation (inflammaging). Macrophage autophagy decreases with age, which is associated with reduced phagocytosis, attenuated oxidative burst, and increased release of pro-inflammatory factors [97]. On the one hand, the defective phagocytic capacity in macrophages leads to inadequate clearance of senescent cells and apoptotic debris, resulting in sustained inflammation. On the other hand, excessive pro-inflammatory factors could further promote osteoclast activation and bone resorption [98]. For instance, IL17 and IFNγ could synergize with RANKL to enhance osteoclast precursor differentiation via TRAF6/ERK/p38 signaling [98]. Additionally, CD38, a bifunctional enzyme that links calcium sensing with metabolic regulation in osteoclasts, emerges as a critical regulator of osteoclastogenesis during aging. Increased CD38 expression in senescent monocytic myeloid-derived suppressor cells (M-MDSCs) enhances their osteoclastogenic potential, contributing to age-related bone loss [32].

Collectively, immunosenescence perturbs skeletal equilibrium by enhancing osteoclast-mediated bone resorption through both adaptive and innate immune dysfunction. Dissecting how discrete immune populations and their cytokine networks converge to control osteoclast fate remains a central challenge for developing targeted therapies that re-establish balanced remodeling in the aging skeleton.

### 3.5. Chondrocytes

Articular cartilage, composed of chondrocytes embedded within a type II collagen and proteoglycan-rich extracellular matrix (ECM), provides low-friction articulation and load distribution in joints [99]. Chondrocytes serve as the sole cellular component responsible for ECM maintenance and turnover, and their proper function is essential for cartilage structural integrity [100]. The progressive deterioration of chondrocyte function and subsequent ECM breakdown are central pathological features of osteoarthritis (OA) and rheumatoid arthritis (RA), both of which are strongly associated with aging [101].

Within the OA joint, macrophages emerge as key mediators of synovial inflammation through dynamic interactions with chondrocytes. These immune cells accumulate in synovial tissue, where they undergo polarization into distinct M1 and M2 subtypes [102]. Immunosenescence drives a phenotypic shift toward M1 dominance, characterized by an increased M1/M2 ratio that correlates with OA progression [102]. M1 macrophages contribute to cartilage pathology through production of catabolic factors (IL1β, IL6, MMP13, ADAMTS5) that promote chondrocyte hypertrophy and matrix degradation while suppressing cartilage matrix genes (*Acan*, *Col2a1*) [103,104]. The interaction between macrophages and chondrocytes establishes a vicious cycle: products released from damaged or dead chondrocytes further activate macrophages, ultimately resulting in joint destruction [105,106].

Recent therapeutic approaches have focused on breaking this cycle by modulating macrophage polarization. Spermidine, an anti-aging natural substance, exerts its effects through anti-apoptosis and anti-inflammation. Instead of directly targeting chondrocytes, it has been demonstrated to increase M2 polarization in synovial macrophages by inhibiting MAPK and NF-κB signaling, and indirectly promote the anabolism and inhibit the catabolism of chondrocytes, thus attenuating cartilage degradation in OA models [107]. Similarly, NAD-loaded hydrogel microspheres (NAD@NPs@HM) have been shown to reprogram synovial macrophage metabolism toward the M2 phenotype, alleviating synovitis and counteracting chondrocyte senescence [108]. These studies highlight the potential of targeting macrophage polarization as a strategy to preserve cartilage integrity in the aging joint.

Beyond macrophages, age-related decline in Treg function also exacerbates cartilage damage. Tregs maintain joint homeostasis through their secretion of anti-inflammatory cytokines (interleukin 10 (IL10), TGFβ), but their functional impairment in aging leads to compromised immunosuppression and uncontrolled inflammation that contributes to both OA and RA pathogenesis [109,110]. Furthermore, the senescence of myeloid cells establishes a chronic low-grade inflammatory state that not only predisposes to cartilage damage but may also facilitate the onset of autoimmune diseases like RA [111]. Collectively, these findings underscore the complex interplay between immunosenescence and chondrocyte dysfunction in age-related joint disorders, highlighting the need for therapeutic strategies that target multiple components of this pathological network.

**Figure 3:**
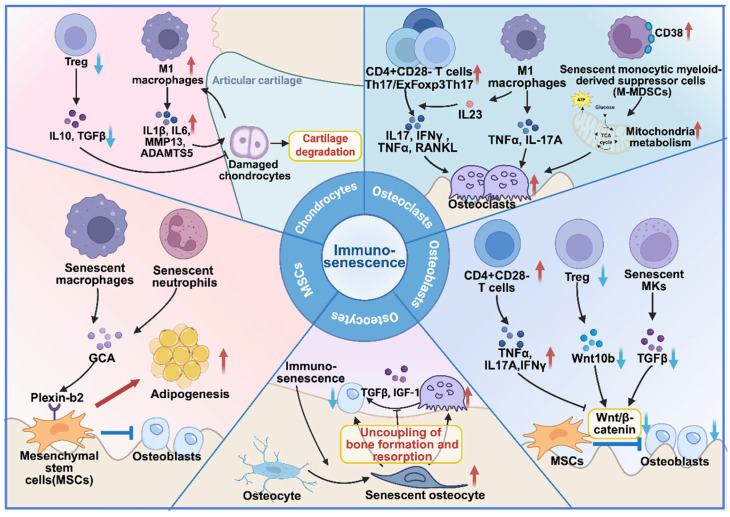
Immunosenescence disrupts bone homeostasis by modulating the functionality of bone-related cells. Immunosenescence affects MSCs, osteoblasts, osteocytes, osteoclasts, and chondrocytes through key regulatory mechanisms, including GCA/Plexin B2 interactions, Wnt/β-catenin signaling, and RANKL/RANK pathways, which collectively govern bone cell maintenance and activity. Red arrows indicate upregulation or increase; blue arrows indicate downregulation or decrease. Black arrows (→) indicate promotion or activation; black T-bars (⊥) indicate inhibition or suppression. Created in BioRender. Dong, B. (2026). Retrieved from https://BioRender.com/wpmvmfg (accessed on 3 April 2026).

In summary, immunosenescence reshapes the bone microenvironment through the accumulation of senescent immune cells and their associated secretory and phenotypic alterations. These interconnected changes converge to create a pro-inflammatory niche that simultaneously impairs MSC osteogenesis, suppresses osteoblast function, drives osteoclast hyperactivation, disrupts osteocyte mechanosensation, and promotes chondrocyte catabolism. This view of the “senescent bone niche” frames immune dysregulation as a common upstream driver of diverse skeletal cell dysfunctions, integrating observations that are often considered in isolation (Figure 3 and Table 2).

## 4. Roles of Immunosenescence on Bone-Related Diseases

### 4.1. Bone Fracture and Bone Defect Healing

Bone fractures and critical-sized bone defects represent clinically significant conditions whose incidence rises dramatically with aging [112,113]. Both types of injury trigger a complex repair process that is profoundly influenced by the functional status of the immune system. Successful restoration of bone continuity requires a precisely orchestrated transition from pro-inflammatory to pro-regenerative immune responses, and immunosenescence disrupts this balance at multiple points.

Macrophages serve as central coordinators of the healing cascade. During early inflammation, M1-polarized macrophages secrete cytokines and chemokines that recruit mesenchymal stem cells [114]. As healing progresses, a transition to M2 macrophages facilitates vascular reconstruction and bone matrix mineralization through release of anti-inflammatory and osteogenic factors [52]. Aging disrupts this transition, creating a persistently pro-inflammatory microenvironment that impairs regeneration [115]. Two recent studies have identified specific molecular defects underlying this age-related macrophage dysfunction. First, aged macrophages exhibit downregulation of TREM2, and TREM2 loss in young mice recapitulates the impaired fracture healing phenotype, identifying TREM2 as a critical regulator of age-related healing deficits [116]. Second, senescent macrophages within the fracture callus secrete grancalcin (GCA), which induces stem cell senescence via the Plexin-B2 receptor, pinpointing a precise SASP factor as a therapeutic target [117].

Beyond macrophages, T cell alterations also contribute to impaired healing. Elevated C-X-C motif chemokine receptor 3 (CXCR3) expression on CD8^+^ T cells may prolong the inflammatory response in aged individuals, and circulating immunosenescent CD8^+^ terminally differentiated effector memory CD8^+^ T cells re-expressing CD45RA (TEMRA) cells have been identified as a prognostic biomarker for impaired fracture healing in a prospective clinical trial [15,118].

Therapeutic strategies have evolved from broad immunomodulation toward more precise targeting of identified pathogenic mechanisms. Approaches that promote M2 polarization—such as local transplantation of young macrophages [119], biodegradable Zn-2Cu–0.5Zr alloys [120], or IL4 delivery [121]—have shown promise in preclinical models. More sophisticated strategies aim to coordinate multiple healing phases sequentially, exemplified by hydrogels that co-deliver a stem cell recruiter and M2 macrophage-derived exosomes [122]. Direct neutralization of GCA using antibody-loaded hydrogels has been shown to rejuvenate bone healing in aged mice, demonstrating the feasibility of targeting specific SASP factors [123].

Collectively, these studies establish that immunosenescence impairs bone repair through defects in macrophage polarization, T cell function, and SASP-mediated inhibition of skeletal progenitors. The identification of discrete molecular targets—triggering receptor expressed on myeloid cells 2 (TREM2), GCA, and specific T cell subsets—provides a basis for developing interventions that restore healing capacity by remodeling the aged immune microenvironment.

### 4.2. Osteoarthritis and Rheumatoid Arthritis

#### 4.2.1. Osteoarthritis (OA)

Osteoarthritis (OA) is a degenerative musculoskeletal disorder characterized by progressive articular cartilage destruction, with typical pathological features including cartilage matrix degradation, osteophyte formation, and periarticular inflammation. Its pathogenesis involves biomechanical, genetic, and biochemical factors that disrupt cartilage homeostasis, with aging serving as a predominant risk factor [101]. This demographic pattern underscores the critical need to elucidate how age-related immunological decline and its associated inflammatory microenvironment contributes to OA pathogenesis.

Immunosenescence is strongly associated with OA progression [124]. Age-related decline in innate and adaptive immune function within joints weakens immune defenses, resulting in persistent low-grade chronic inflammation. This process drives the accumulation of senescent cells and enhances the secretion of SASP in joint tissues (cartilage, synovium, and subchondral bone), increasing susceptibility to OA. Lan et al. have characterized this relationship by identifying distinct senescence-associated immune phenotypes in OA, classifying them into two molecular subtypes—immune-activated and immune-suppressed—based on specific biomarkers (e.g., *CACNA1A*, *FLT1*, *KCNAB3*) [125]. These findings reveal actionable immunotherapeutic targets for OA, enabling precision strategies to modulate pathogenic immune responses and improve clinical outcomes through immune homeostasis restoration.

Evidence indicates that macrophages play a central role in OA. Immunosenescence disrupts cartilage homeostasis by promoting a pro-inflammatory M1 macrophage polarization [102]. M1 macrophages overproduce cytokines and MMPs that drive cartilage degradation [103,126]. Bondeson et al. demonstrated that macrophage depletion in co-cultures with OA patient-derived synovial cells significantly reduced pro-inflammatory cytokines (e.g., IL1, IL6, and TNFα) and MMPs (MMP1/3/9/13), thereby attenuating inflammatory responses and OA pathogenesis [126]. Damage-associated molecular patterns (DAMPs) released from injured cartilage activate synovial macrophages [106], creating a vicious cycle of inflammation and matrix breakdown [127].

Parallel to macrophage-driven inflammation, immunosenescence remodels the B cell compartment in OA. Multi-omics studies identify aging-specific, metabolically distinct B cell clusters that exhibit enhanced activation and are implicated in OA progression, concurrently yielding age-associated peripheral blood biomarkers (MAPK1, MAP3K8, ING1, LDLR, and NUP153) [128].

Emerging senomorphic therapies targeting SASP components—including NF-κB, JAK/STAT, and particularly MMP13—show promise in modulating OA progression by simultaneously addressing inflammatory and catabolic pathways. Although clinical trials of single cytokine inhibition (TNFα, IL1β) have been disappointing [129,130], preclinical studies indicate that selective MMP13 blockade preserves cartilage integrity [131]. The context-dependent roles of SASP factors underscore the need for precision approaches based on OA endotypes, focusing on dominant SASP drivers and combinatorial regimens.

#### 4.2.2. Rheumatoid Arthritis (RA)

Rheumatoid arthritis (RA) is a systemic autoimmune disease characterized by chronic erosive polyarthritis, with hallmark pathological features including abnormal synovial hyperplasia, pannus formation, and progressive destruction of articular cartilage and subchondral bone. These pathological changes ultimately result in joint deformity and functional impairment [132]. The age-dependent increase in RA incidence strongly implicates immunosenescence as a contributing factor that exacerbates disease severity through immune dysfunction and bone homeostasis disruption [133].

The adaptive immune system, particularly T cell senescence, plays a critical role in RA progression. Senescent T cells exhibit characteristic phenotypic changes including telomere shortening, DNA damage accumulation, and metabolic reprogramming that lead to cell cycle arrest and the secretion of SASP [1]. They release pro-inflammatory cytokines (IL6, TNFα, IL1β, IL17) that activate RANKL signaling and suppress Wnt/β-catenin, driving periarticular and systemic bone loss [134,135]. Additionally, senescent T cells upregulate C-X3-C motif chemokine receptor 1 (CX3CR1), promoting inflammatory cell recruitment [136], and skew toward Th17 rather than Treg differentiation, aggravating synovial inflammation [137]. Currently, the therapeutic strategies for RA have evolved significantly with the development of targeted biologic and small-molecule therapies. First, monoclonal antibodies, particularly TNFα and IL17A, exert their effects by directly targeting proinflammatory cytokines and interrupting their downstream signaling cascades [138]. Second, immunomodulatory strategies employing low-dose IL2 demonstrate clinical efficacy through preferential expansion of Tregs, thereby rebalancing the Th17/Treg axis that is characteristically dysregulated in RA [139]. Third, Janus kinase (JAK) inhibitors exhibit multimodal therapeutic effects by promoting osteogenesis and inhibiting osteoclastogenesis, collectively contributing to their clinical benefits in RA [140].

Innate immunosenescence also contributes to RA pathogenesis [141]. An expansion of CD14^bright^/CD56^+^ monocytes with enhanced inflammatory potential (TNF, IL10, IL23, reactive oxygen intermediates) has been observed in RA patients [142]. Moreover, senescent macrophages promote synovial fibroblast invasiveness via an HK3-lactate metabolic-epigenetic axis [143]. The clinical correlation between reduction of this subset following anti-TNF therapy and disease improvement suggests its potential involvement in immunosenescence-related disease mechanisms [142], though further research is needed to fully elucidate these relationships.

### 4.3. Osteoporosis

Osteoporosis is a systemic skeletal disorder characterized by compromised bone microarchitecture, reduced bone mineral density, and increased fracture risk, arising from an imbalance where bone resorption outpaces formation [144]. Aging, estrogen deficiency, and metabolic disorders are well-established risk factors [144], but emerging evidence highlights immunosenescence as a critical driver of this remodeling imbalance.

The concept of “immunoporosis” emphasizes the role of chronic low-grade inflammation in age-related bone loss [145]. With age, senescent macrophages and neutrophils accumulate in bone marrow and inhibit osteogenesis by promoting MSC adipogenesis. Macrophage polarization shifts toward the pro-inflammatory M1 phenotype, which dominates the bone microenvironment and secretes bone-resorptive cytokines [146]. In postmenopausal osteoporosis, estrogen deficiency synergizes with immunosenescence, further elevating the M1/M2 ratio and sustaining pro-inflammatory cytokine production [147]. Concurrently, bone marrow monocytes from aged mice acquire enhanced pro-osteoclastic capacity through epigenetic reprogramming, such as DNA demethylation of the IL19 promoter, driving bone loss independently of gonadal aging [33]. Interventions targeting this polarization imbalance show promise: the prebiotic 2′-fucosyllactose (2′-FL) reduces M1 macrophage subsets in aged mice and mitigates bone loss [148], while functional Fe_3_O_4_ nanoparticles enhance mitochondrial quality and reprogram senescent macrophages toward a pro-regenerative M2 phenotype, promoting bone formation [149].

Immunosenescence also involves progressive dysregulation of T cell subsets. The Th17/Treg ratio increases with age, creating a low-grade inflammatory milieu through excessive cytokine production [150]. Restoring Th17/Treg balance suppresses osteoclastogenesis and bone resorption, highlighting this immunomodulatory approach as a promising therapeutic strategy [151]. Beyond T cell dysregulation, evidence also implicates the B_ACSM3 subset in osteoporotic bone loss in that this age-associated B cell population, expanded in osteoporosis, promotes osteoclastogenesis via the transcription factor MYB, presenting a potential therapeutic target within immunoporosis [152]. 

Collectively, these findings establish that immunosenescence drives osteoporosis through parallel defects in innate and adaptive immunity—macrophage polarization imbalance, Th17/Treg dysregulation, and B cell-mediated osteoclastogenesis—that collectively remodel the senescent bone niche. Targeting these immune pathways may complement existing treatments by addressing the underlying immune dysregulation that perpetuates bone loss in aging populations.

### 4.4. Intervertebral Disc Degeneration

Intervertebral disc degeneration (IVDD) is a chronic disorder characterized by progressive ECM degradation, annulus fibrosus disruption, and nucleus pulposus (NP) dysfunction, representing a leading cause of low back pain [6,153]. Current therapies address symptoms rather than halting disease progression, underscoring the need to understand underlying mechanisms [154]. Emerging evidence implicates dysregulated immune responses and chronic inflammation in IVDD initiation and progression [155].

Under physiological conditions, nucleus pulposus cells (NPCs) reside in an immune-privileged milieu shielded by the blood-NP barrier [156]. Barrier disruption permits infiltration of macrophages and T cells, triggering a pro-inflammatory cascade [157]. Infiltrating immune cells release TNFα, IL1α/β, and IL6, which enhance ECM catabolism and promote NPC senescence and apoptosis, accelerating IVDD [158]. Immunosenescence amplifies this process through two key mechanisms: dysregulated macrophage polarization and aberrant T cell differentiation [6,155].

Immunosenescence shifts macrophage polarization toward the pro-inflammatory M1 phenotype. M1-derived exosomes transport lipocalin2 (LCN2) to NPCs, activating NF-κB signaling, triggering cellular senescence, and exacerbating ECM degradation—a dual mechanism that drives IVDD progression [31].

Parallel to macrophage alterations, immunosenescence drives pathological hyperactivation of Th17 cells. IL17, the signature Th17 cytokine, upregulates matrix-degrading enzymes (ADAMTS7, MMP3, MMP13) [159,160], amplifies inflammatory responses in NPCs via NF-κB and AP-1 signaling [161,162], and initiates a self-perpetuating cycle where chronic inflammation accelerates senescence, which in turn perpetuates inflammation.

The growing recognition of immune involvement in IVDD has spurred investigation into immune-metabolic crosstalk, revealing potential therapeutic targets. Strategies aimed at modulating these immune-mediated degenerative processes may offer disease-modifying approaches that address root causes rather than symptoms [163].

### 4.5. Periodontitis

Periodontitis is a chronic inflammatory disease initiated by oral microbiota dysbiosis, characterized by progressive alveolar bone loss [164]. While microbial infection is the primary etiological factor, aging exacerbates disease progression by disrupting immune homeostasis [165].

Adaptive immunosenescence impacts periodontal health through multiple mechanisms. Immunosenescence drives CD4^+^ T cell dysfunction and accumulation of senescent CD4^+^ T cells that bias toward Th17 differentiation, while Treg function is impaired via the DCAF1/GSTP1/ROS axis [166], creating a Th17/Treg imbalance [167]. Elevated IL17 synergizes with RANKL to amplify osteoclast differentiation and alveolar bone resorption [98]. Immunosenescence also enhances gingival follicular helper T (Tfh) cell responses, altering T cell-mediated B cell responses [168] and reduces anti-Porphyromonas gingivalis IgG levels, reflecting declining humoral immunity [169]. Therapeutic strategies targeting the dysregulated adaptive immune response have demonstrated significant potential. Resveratrol has been shown to ameliorate periodontitis by reducing both the Th17/Th2 ratio and RANKL expression [170]. Furthermore, direct IL17 neutralization inhibits alveolar bone loss and osteoclast activity in murine models, while blockade of upstream regulators (IL6R and IL23) similarly attenuates periodontal destruction by suppressing Th17 activation [171,172]. Collectively, these approaches establish the adaptive immune system as a key therapeutic target for mitigating periodontitis-associated bone loss through osteoimmunomodulation.

Innate immunosenescence actively contributes to periodontitis pathogenesis. In aged murine models, gingival tissues demonstrate elevated levels of pro-inflammatory cytokines (IL1β, TNFα, IL17) and upregulation of innate immune receptors (TLR2, CD14, CD11b, CD18, C5aR, and TREM3) [173], which amplify inflammatory responses and promote periodontal bone loss via NF-κB and RANKL signaling [174]. TLR9 has been identified as a critical amplifier of periodontal inflammaging, fostering local SASP and alveolar bone loss through enhanced DAMP (S100A8/A9) signaling and an elevated p16INK4a/p19ARF ratio [175]. However, Akkaoui et al. reveal that the pro-osteoclastogenic response via the *P. gingivalis* LPS/TLR4 pathway is attenuated with age, illustrating the complexity of innate immune remodeling during immunosenescence [176]. Colony-stimulating factor 1 receptor (CSF1R) signaling promotes glycolytic reprogramming in macrophages, driving their senescence; therapeutic CSF1R inhibition reverses this shift and alleviates periodontitis, highlighting immunometabolism as a novel target [177]. Impaired dendritic cell migration via the advanced glycation end products (AGE)- receptor for AGE (RAGE) axis and reduced oral microbial diversity jointly facilitate *P. gingivalis* expansion, provoking heightened adaptive responses that exacerbate bone destruction [178].

Both adaptive and innate immunosenescence may stem from systemic immune dysregulation such as clonal hematopoiesis driven by DNMT3A mutations, which induces multi-lineage dysfunction—impaired Treg suppression, enhanced Th17 responses, defective macrophage efferocytosis, and exacerbated IL17/neutrophil-driven inflammation—collectively exacerbating periodontal bone loss [179]. Concurrently, immunosenescence induces significant alterations in oral microbial composition, characterized by increased abundance of *Fusobacterium*, *P. gingivalis*, *F. alocis*, *Pasteurellaceae*, and *Prevotella* [180]. These microbial shifts promote pro-inflammatory gene expression and dysregulated immune responses, further disrupting bone homeostasis [181]. Systemic strategies targeting age-related immune dysregulation, such as baicalin, have shown efficacy in mitigating periodontal bone loss in aged models [182].

**Figure 4:**
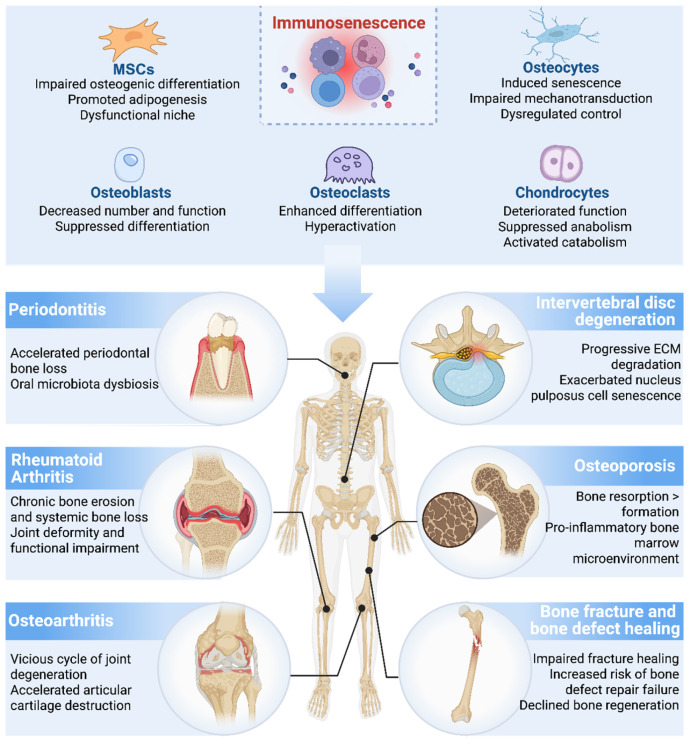
Schematic diagram of the major immunosenescence-driven mechanisms in bone-related diseases. In bone-related diseases, immunosenescence enhances inflammatory responses, promoting osteoclastogenesis and suppressing osteogenesis, thereby leading to the disruption of bone homeostasis, articular cartilage degradation, periodontal bone loss, and degenerative changes in the intervertebral disc ECM. Created in BioRender. Dong, B. (2026). Retrieved from https://BioRender.com/t2e8e6i (accessed on 3 April 2026).

The major skeletal disorders discussed in this section and their associated therapeutic strategies are summarized in Figure 4 and Table 3, respectively.

## 5. Conclusions and Future Perspectives

Immunosenescence profoundly disrupts bone homeostasis through the accumulation of senescent immune cells and their associated secretory and phenotypic alterations, which collectively reshape the bone marrow microenvironment [183]. Accumulating evidence demonstrates that these interconnected changes converge to create a pro-inflammatory niche that simultaneously impairs MSC osteogenesis, suppresses osteoblast function, drives osteoclast hyperactivation, disrupts osteocyte mechanosensation, and promotes chondrocyte catabolism. The concept of the “senescent bone niche” provides a unifying framework for understanding how immune dysregulation acts as a common upstream driver of diverse skeletal pathologies—including osteoporosis and osteoarthritis as prototypes of systemic and local bone loss, respectively, as well as delayed fracture healing, intervertebral disc degeneration, and periodontitis as paradigms of impaired regenerative and defensive responses.

Despite these advances, several critical knowledge gaps remain. First, distinguishing the effects of intrinsic cellular aging from those of accumulated immunological memory on bone homeostasis remains technically challenging. Most current animal models fail to recapitulate the complexity of human immunosenescence, where decades of antigen exposure shape the immune repertoire. Second, while immune cells have been extensively studied, the role of bone-embedded cells—particularly osteocytes—as active participants in the senescent immune dialogue is largely unexplored. Osteocytes’ capacity to sense and respond to SASP factors, and whether they themselves contribute to the SASP pool, represents a fertile ground for investigation. Third, the heterogeneity of the SASP across different immune cell subsets and disease contexts poses a significant barrier to therapeutic development. Identifying which SASP components are causally linked to specific skeletal outcomes, rather than merely correlative, is essential for precision targeting.

Emerging therapeutic strategies are moving beyond broad immunosuppression toward precise intervention. Senolytic agents that selectively clear senescent immune cells, and senomorphic agents that modulate their secretory phenotype, have shown promise in preclinical models. The identification of specific targets such as GCA, TREM2, and CSF1R exemplifies this shift [35,121,184,185]. Notably, early evidence has emerged from a placebo-controlled trial in older adults, in which low-dose rapamycin reduced p21, a marker of DNA damage-induced senescence, in circulating immune cells [186]. Nevertheless, large-scale longitudinal immunophenotyping studies that track immune and skeletal parameters in aging populations are urgently needed to establish causal relationships and identify optimal intervention windows.

To overcome the limitations of animal models and accelerate clinical translation, advanced human-relevant platforms offer promising alternatives. Bone marrow organoids are three-dimensional, self-assembled structures derived from human iPSCs or mesenchymal/hematopoietic co-cultures. They recapitulate key components of the human bone marrow niche, including stromal cells, myeloid cells, and terminally differentiated CD57^+^ NK cells, enabling the study of immune–stromal interactions in a human system [187,188]. Bone-on-a-chip platforms, a type of microphysiological system (MPS), integrate microfluidic channels with living tissues to enable real-time monitoring of immune–stromal crosstalk under dynamic mechanical loading [189]. Importantly, MPS can reproduce hallmarks of human aging (e.g., age-related gene expression shifts and oxidative DNA damage) within days, allowing rapid testing of anti-aging interventions [190]. The integrated bone/cartilage organoid-on-chip (BCoC) platform combines structural fidelity with microenvironmental controllability [191]. Complementing these in vitro platforms, humanized mouse models reconstitute a human immune system in vivo and have recently been used to recapitulate age-related T cell senescence, providing a complementary system to validate senolytic candidates in a living organism with a human immune repertoire [192]. In the context of the senescent bone niche, these human-derived platforms offer a more complex and physiologically relevant environment than traditional animal models by avoiding the epigenomic and genomic differences that hinder cross-species translation. They can recapitulate complex organ functions in vitro, thereby providing a more predictive tool for studying human immunosenescence and age-related skeletal disorders. However, their direct application to study the senescent bone niche and to test senolytic or senomorphic interventions in the context of immune–bone crosstalk remains largely unexplored. Current human-relevant platforms face remaining limitations: they fail to recapitulate the full immune repertoire of aged bone marrow, lack a standardized engineering roadmap for generating reproducible adult-like organoids, rely on endpoint analyses that cannot capture dynamic aging-related cellular responses, and predominantly operate at the single-organ level, missing the multi-system crosstalk that drives systemic aging. Future investigations should therefore focus on integrating aged human immune cells into these platforms, validating their predictive capacity for skeletal outcomes, and systematically comparing platform outputs with clinical phenotypes. Such efforts will be essential to determine whether these models can ultimately guide the rational design of combination therapies targeting multiple nodes of the senescent bone niche.

In conclusion, the framework of the “senescent bone niche” reframes skeletal aging not as a collection of cell-autonomous deficits, but as a systems-level failure of immune-stromal crosstalk. Addressing the knowledge gaps outlined above—through integrated approaches combining human cohorts, advanced models, and precision therapeutics—holds the potential to transform the management of age-related skeletal diseases by restoring a youthful immune microenvironment and, with it, bone homeostasis.

## Figures and Tables

**Table 1:** Overview of immunosenescence-related alterations in immune cell subsets.

Immune Cell Subset	Category	Specific Change	References
Macrophages	Subset Distribution	M1/M2 ratio ↑	[35]
	Functional Changes	Antigen-presenting function ↓; Phagocytic activity ↓	[12]
	Surface Marker	MHC class II ↓, TLRs ↓	[12]
NK cells	Subset Distribution	NK cell percentage (CD3^−^CD56^+^CD16^+^) ↑; CD56^dim^/CD56^bright^ ratio ↑	[36]
Dendritic Cells	Subset Distribution	cDC1, cDC2 percentage ↓; pDC percentage (trend) ↓	[37]
	Functional Changes	Phagocytic activity (cDC2) ↓	[37]
	Surface Marker	CD32^+^ cDC2 ↓	[37]
Neutrophils	Subset Distribution	Neutrophil count (myeloid bias) ↑	[38]
	Functional Changes	Apoptosis ↓	[39]
	Metabolic Changes	mtDNA leakage ↑; Glycolysis ↑ (Hif1α ↑)	[38,39]
T cells	Subset Distribution	Naïve T cells ↓; CD28^−^ memory T cells ↑	[40]
	Cytokines	TNFα, IL17A, IL6, IL1β, IFNγ ↑	[40,41]
	Surface Marker	CD28, CD27 ↓; p16, CD57, KLRG1 ↑; PD1, TIM3 ↑	[40,42,43,44]
B cells	Subset Distribution	Naïve B cells ↓; Aging-associated B cells (ABCs) ↑	[45,46]
	Cytokines	TNFα (ABCs) ↑; S100A8/A9 (senescent B cells) ↑	[46,47]
	Surface Marker	CD11c (ABCs) ↑	[48]
	Epigenetic Changes	Large-scale 3D chromatin reorganization silences key regulators such as Ebf1	[49]
All immune cells		Telomere length ↓; SA-β-gal ↑	[34,50]

“↑”: enhanced; “↓”: impaired.

**Table 2:** Effects, Mechanisms, and Interventions Related to Immunosenescence of Bone-Related Cells.

Target Skeletal Cell	Model System	Immunosenescent Cell and State	Key Molecules	Signaling Pathways	Impact on Bone/Cartilage Homeostasis	Potential Interventions and Therapeutic Effects	References
MSCs/Osteoprogenitors	Mouse calvarial defect	Macrophages (Aged)	dimethyl itaconate (DMI)	NA	Osteogenic differentiation ↓	DMI-releasing material: Induced M2 polarization and mitochondrial transfer, improved repair	[70]
	Mouse calvarial defect	Macrophages (Pro-inflammatory/M1)	IL6, IL1β, TNFα	mTOR/Autophagy	Osteogenic differentiation ↓, Bone regeneration impaired	Rapamycin-loaded silica nanocarriers (R@HSNs): Targeted delivery to macrophages induces autophagy-mediated M2 polarization, promoting osteogenesis and bone repair	[71]
	Aged rat calvarial defect	Macrophages (Aged, SASP ↑)	ROS; SASP factors	Nrf2	BMSC senescence ↑, Osteogenesis ↓	3D-bioprinted Mg-Ce-MOF scaffolds: Scavenge ROS, reduce SASP, release Mg^2+^ to activate Nrf2, delay BMSC senescence, and promote M2 macrophage polarization	[72]
	Aged mouse model	Macrophages (Aged, SASP ↑)	Grancalcin (GCA)	Plexin B2	Osteogenesis ↓; Adipogenesis ↑	GCA-neutralizing antibodies: Enhanced healing in aged models	[30]
Osteoblast	Aged mouse calvarial defect model	Tregs (Senescent)	Progranulin (PGRN) ↓	PGRN/EGFR/PI3K/AKT	Osteogenic induction of osteoblast precursors impaired	Recombinant PGRN (rPGRN) supplementation: Restored the osteo-inductive capacity of senescent Tregs	[80]
	Aged mouse model	Megakaryocytes (Aged)	TGFβ ↓	NA	Osteoblast proliferation and differentiation ↓	TGFβ restoration: Stimulated osteoblast precursor activity	[82]
Osteoclasts	Human RA/OP patients	Senescent T cells (CD4^+^CD28^−^ & CD4^+^CD28^−^FoxP3^+^ subsets)	RANKL, TNFα ↑	RANKL/RANK/NFATc1	Osteoclastogenesis ↑, Bone loss ↑	RANKL inhibition; Anti-TNF therapy	[29]
	Ligature-induced periodontitis rat model	Immune cells (Inflammaging)	IL17, IFNγ ↑	RANKL/NFATc1	Enhances osteoclastogenesis (RANKL-dependent), exacerbates alveolar bone loss	Neutralizing IL17/IFNγ: Reduced inflammation and bone loss	[98]
	Aged mouse model	M-MDSCs (Aged)	CD38 ↑	Metabolic reprogramming (↑mitochondrial respiration & glucose metabolism)	Osteoclastogenic potential ↑	CD38 inhibitor (78c): Reduced metabolic hyperactivity and inhibited osteoclastogenic potential of aged M-MDSCs	[32]
Chondrocytes and Cartilage	Mouse trauma- and aging-induced OA model	Synovial Macrophages (Senescent, M1)	SASP factors	p53/senescence pathway; STAT3/ADAM17/MerTK	Cartilage degeneration ↑	Senotherapeutic nanoparticle (pCQ/SOD): Clears senescent macrophages, promotes M2 repolarization, alleviates synovitis and cartilage degradation	[102]
	Mouse DMM-induced OA model	Synovial Macrophages (M1)	NA	ERK/NF-κB (in macrophages)	Cartilage degradation ↑	Spermidine (SPD) treatment: Inhibits ERK/NF-κB in macrophages, promotes M2 polarization, and indirectly protects cartilage	[107]
	Aged mouse model	Synovial Macrophages (Aged, metabolic dysfunction)	NA	Metabolic reprogramming	Disrupts chondrocyte metabolic balance, promotes catabolism, exacerbates synovitis	NAD^+^-loaded lubricated hydrogel microspheres (NAD@NPs@HM): Reprograms macrophage metabolism to promote M2 repolarization, alleviating synovitis and cartilage catabolism	[108]

“↑”: enhanced; “↓”: impaired.

**Table 3:** Immunosenescence in Bone-Related Diseases: Effects, mechanisms and interventions.

Disease	Model System	Immunosenescent Cell Type	Key Molecules	Signaling Pathways	Effector Bone/Cartilage Cell	Impact on Bone Homeostasis	Potential Interventions and Therapeutic Effects	References
Fracture/Bone Defect	Aged mouse fracture model; Trem2 KO mouse	Macrophages (Aged, pro-inflammatory)	TREM2 ↓	NA	Healing microenvironment and osteoprogenitors	Impaired fracture healing, inflammatory dysregulation	Targeting TREM2: Potential to restore macrophage function and improve healing (based on KO phenotype)	[116]
	Young vs. aged rat osteotomy model	Macrophages (Aged, M2 polarization ↓)	M2 macrophage markers (e.g., CD206) ↓	NA	Angiogenesis and subsequent osteogenesis	Angiogenesis ↓, Bone regeneration ↓, Fibrosis ↑	Local transplantation of CD14^+^ macrophage precursors: Partially rescued bone regeneration and angiogenesis in aged rats	[119]
	Aged osteoporotic rat tibial fracture model	Macrophages (Aged, M1-prone microenvironment)	NA	Wnt/β-catenin	BMSCs, Osteoclasts	Bone regeneration impaired	Biodegradable Zn-2Cu-0.5Zr alloy implants: Release Zn^2+^ to promote macrophage M2 polarization, creating a pro-osteogenic immune microenvironment and improving fracture healing	[120]
	Aged mouse fracture model; myeloid-specific Gca knockout	Macrophages (Senescent)	GCA	Plexin-B2/Arg2-mediated mitochondrial dysfunction	Skeletal Stem/Progenitor Cells (SSPCs)	SSPC senescence ↑, Fracture healing impaired	GCA-neutralizing antibody (GCA-NAb): Enhanced fracture healing in aged mice	[117]
	Aged rat cranial defect model (with bone substitute)	Macrophages (Aged, M1 phenotype ↑)	NLRP3 inflammasome (activated)	JAK/STAT6 (in macrophages)	Osteoclasts (via NLRP3); Osteoprogenitors	Bone regeneration impaired	Local IL4 delivery: Promotes M2 macrophage polarization via JAK/STAT6, suppresses NLRP3 inflammasome activation, and improves bone regeneration in aged rats	[35]
	Aged mouse tooth extraction socket healing model	Macrophages (Senescent, within injury site)	GCA	PlxnB2-Arg2 axis (mitochondrial dysfunction)	BMSCs	BMSC senescence ↑, Jawbone healing impaired	GelMA hydrogel delivering GCA-neutralizing antibody (GCA-NAb): Enhanced jawbone healing in aged mice	[123]
Osteoarthritis (OA)	Human OA patients (multi-omics and validation)	B cells (Aged/disease-specific subsets, activated)	NA	Altered differentiation & metabolic reprogramming	Chondrocytes	Cartilage damage ↑	1. Blood biomarkers (MAPK1, MAP3K8, ING1, LDLR, NUP153): Non-invasive diagnostic tool, especially for elderly OA. 2. Targeting age-specific B cell subsets: Potential immunomodulatory strategy for elderly OA.	[128]
	Human LORA patients and healthy controls (blood, LN, synovium)	CD4^+^ T cells (CX3CR1^+^ cytotoxic, age-associated ‘ThA’ subset)	CX3CR1, Granzyme B (GZMB)	NA	Synovial tissue and joint structures	Synovitis ↑, Joint destruction ↑	1. Anti-TNF/IL6 therapy; Targeting CX3CR1 (e.g., E6011). 2. Avoid abatacept. Potential to reduce disease activity in LORA and addresses a treatment-resistant subset (PD-1^+^CD38^+^).	[136]
Rheumatoid Arthritis (RA)	H_2_O_2_/BLM-induced senescent macrophage model; Mouse synovial fibroblast (MSF) co-culture	Macrophages (Senescent, M1-polarized)	IL17 ↑	HK3-mediated histone H3K14 lactylation	Synovial Fibroblasts	Synovial fibroblast proliferation and invasion ↑, joint destruction ↑	Targeting senescent macrophages via HK3 knockdown: Reduces H3K14 lactylation and M1 polarization, thereby inhibiting synovial fibroblast activation.	[143]
	Natural aging mice; LPS-stimulated RAW 264.7 cells	Macrophages (Primed by gut dysbiosis and barrier disruption, M1 phenotype ↑)	TLR4	TLR4/MyD88/NF-κB	Osteoclasts; Osteoblasts	Bone resorption ↑, Bone formation ↓	2′-Fucosyllactose (2′-FL) dietary intervention: Inhibits TLR4/MyD88/NF-κB signaling, reduces M1 macrophage polarization, restores gut microbiota homeostasis, and ameliorates bone loss in aged mice.	[148]
Osteoporosis	Bone marrow monocytes (BMMs) from old mice	Osteoclast precursors (Aged BMMs)	IL19 ↑	Epigenetic regulation	Osteoclasts	Osteoclast differentiation ↑, bone loss ↑	Targeting IL19 or its epigenetic regulation: e.g., increasing IL19 promoter methylation to reduce IL19 expression and inhibit osteoclastogenesis.	[33]
	Aged osteoporotic mouse model; KGM-PEG-SPIONs-functionalized scaffolds	Macrophages (Senescent, mitochondrial dysfunction)	NA	Autophagy activation and Fe–S cluster biogenesis	BMSCs	BMSC osteogenic differentiation ↓, bone formation impaired	Functional Fe_3_O_4_ nanoparticles (KGM-PEG-SPIONs): enhance mitochondrial quality, reprogram senescent macrophages toward M2	[149]
Intervertebral Disc Degeneration (IVDD)	Rat acupuncture IVDD model; LPS-induced NPC degeneration model	Macrophages (M1-polarized)	Lipocalin-2 (LCN2)	LCN2/NF-κB	Nucleus Pulposus Cells (NPCs)	NPC senescence ↑, ECM degradation ↑, IVDD progression ↑	GW4869 (exosome inhibitor): Blocks M1 macrophage-derived exosomal LCN2 to inhibit NF-κB-mediated NPC senescence	[31]
	Human degenerated disc tissue; Cultured human nucleus pulposus (NP) cells	T cells (Th17)	IL17	NF-κB	NPCs	ECM degradation ↑ (Collagen II, Aggrecan ↓), Inflammation ↑	Pioglitazone (PPAR-γ agonist): Activates PPAR-γ to inhibit NF-κB signaling, suppressing inflammation and ECM catabolism in NP cells	[161]
Periodontitis	Ligature-induced periodontitis mouse model; Pg-LPS-stimulated RAW264.7 cells	Macrophages (Senescent)	SASPs	CSF-1R-mediated glycolytic reprogramming	Osteoclasts	Alveolar bone resorption ↑	Pexidartinib (PLX3397): Inhibits CSF-1R, suppresses glycolysis, and reduces macrophage senescence, thereby attenuating periodontal bone loss.	[177]
	Clinical samples; Young/aged WT and TLR9^−^/^−^ mice; BMDMs ex vivo	Macrophages (within an inflammaging/senescence microenvironment)	TLR9, S100A8/A9	NF-κB, p16INK4a/p19ARF senescence axis	Osteoclasts	Alveolar bone resorption ↑	Targeting TLR9 (e.g., inhibitors or genetic ablation): Attenuates inflammaging/senescence and alveolar bone resorption in aged subjects.	[175]
	Dnmt3aR878H/^+^ bone marrow transplantation mouse model	Dysfunctional immune cells (e.g., Tregs, Th17, neutrophils) due to CHIP	IL17 ↑	Epigenetic reprogramming, mTOR signaling	Osteoclasts	Alveolar bone loss ↑; Arthritis bone erosion ↑	Rapamycin (mTOR inhibitor): Inhibits clonal expansion and inflammatory bone loss.	[179]

“↑”: enhanced; “↓”: impaired.

## Data Availability

No new data were created or analyzed in this study. Data sharing is not applicable to this article.
